# Betacoronaviruses SARS-CoV-2 and HCoV-OC43 infections in IGROV-1 cell line require aryl hydrocarbon receptor

**DOI:** 10.1080/22221751.2023.2256416

**Published:** 2023-09-06

**Authors:** Meisam Yousefi, Wai Suet Lee, Wharton O. Y. Chan, Wei He, Marcus G. Mah, Cythia Lingli Yong, Joshua M. Deerain, Lijin Wang, Camille Arcinas, Biaoguo Yan, Dewei Tan, Wan Rong Sia, Akshamal M. Gamage, Jinxuan Yang, Alan Chen-Yu Hsu, Shang Li, Martin Linster, Xinglou Yang, Sujoy Ghosh, Danielle E. Anderson, Gavin J. D. Smith, Chee Wah Tan, Lin-Fa Wang, Yaw Shin Ooi

**Affiliations:** a Program in Emerging Infectious Diseases, Duke-NUS Medical School, Singapore, Singapore; b Victorian Infectious Diseases Reference Laboratory, The Peter Doherty Institute for Infection and Immunity, Melbourne, Australia; c Centre for Computational Biology, Duke-NUS Medical School, Singapore, Singapore; d Yunnan Key Laboratory of Biodiversity Information, Kunming Institute of Zoology, Chinese Academy of Sciences, Kunming, People’s Republic of China; e Immune Health Research Program, Hunter Medical Research Institute, New Lambton Heights, Australia; f College of Health, Medicine and Wellbeing, The University of Newcastle, Callaghan, Australia; g Program in Cancer and Stem Cell Biology, Duke-NUS Medical School, Singapore, Singapore; h Program in Cardiovascular and Metabolic Disorders, Duke-NUS Medical School, Singapore, Singapore; i Department of Microbiology and Immunology, The University of Melbourne, The Peter Doherty Institute for Infection and Immunity, Melbourne, Australia; j Infectious Diseases Translation Research Program, Department of Microbiology and Immunology, Yong Loo Lin School of Medicine, National University of Singapore, Singapore, Singapore; k Infectious Diseases Labs, Agency for Science, Technology and Research (A*STAR), Singapore, Singapore

**Keywords:** IGROV-1, COVID-19, betacoronavirus, SARS-CoV-2, HCoV-OC43, Genome-scale CRISPR screening, AHR, DiMNF

## Abstract

The emergence of novel betacoronaviruses has posed significant financial and human health burdens, necessitating the development of appropriate tools to combat future outbreaks. In this study, we have characterized a human cell line, IGROV-1, as a robust tool to detect, propagate, and titrate betacoronaviruses SARS-CoV-2 and HCoV-OC43. IGROV-1 cells can be used for serological assays, antiviral drug testing, and isolating SARS-CoV-2 variants from patient samples. Using time-course transcriptomics, we confirmed that IGROV-1 cells exhibit a robust innate immune response upon SARS-CoV-2 infection, recapitulating the response previously observed in primary human nasal epithelial cells. We performed genome-wide CRISPR knockout genetic screens in IGROV-1 cells and identified Aryl hydrocarbon receptor (AHR) as a critical host dependency factor for both SARS-CoV-2 and HCoV-OC43. Using DiMNF, a small molecule inhibitor of AHR, we observed that the drug selectively inhibits HCoV-OC43 infection but not SARS-CoV-2. Transcriptomic analysis in primary normal human bronchial epithelial cells revealed that DiMNF blocks HCoV-OC43 infection via basal activation of innate immune responses. Our findings highlight the potential of IGROV-1 cells as a valuable diagnostic and research tool to combat betacoronavirus diseases.

## Introduction

Coronavirus disease 2019 (COVID-19) is one of the most severe and best documented pandemics of the modern human history. As of March 2023, Severe Acute Respiratory Syndrome Coronavirus 2 (SARS-CoV-2), has infected at least 600 million individuals worldwide resulting in nearly seven million deaths (https://coronavirus.jhu.edu/). Efforts to mitigate the impact of the pandemic occurred at an unprecedented pace, with research endeavours focused on vaccines and antiviral therapeutic development beginning almost immediately after the isolation and sequencing of the virus. Human primary airway cells were first used to culture the virus [1], but given the limitations associated with the primary cell culture systems, other cell lines have been routinely utilized to propagate the virus *in vitro* in both clinical diagnostics and research laboratories [2]. The African green monkey Vero cell line and its derivatives (e.g. Vero E6) are one of the most common cell lines used for SARS-CoV-2 research, owing to their favourable attributes such as availability and ease of culture, supporting production of high viral titre stocks, and observable cytopathic effects (CPE) following infection. However, using these cells for SARS-CoV-2 research can pose significant drawbacks. During replication in these non-human origin cells, the virus acquires adaptative mutations resulting in key alterations such as the loss of the furin cleavage site [3, 4]. Furthermore, SARS-CoV-2 cellular host factors differ between human and non-human cells, an issue highlighted when assessing the potential effectiveness of chloroquine as a host-directed antiviral against SARS-CoV-2 [5, 6]. Multiple human-derived cell lines have been utilized for the propagation of SARS-CoV-2, including the naturally susceptible cell lines, e.g. Calu-3, Huh7, and Caco-2, as well as genetically engineered cell lines such as 293T and A549 cells that require the overexpression of the virus receptor, angiotensin converting enzyme 2 (ACE2). A consensus has yet to be reached on the optimal cell line for the study of SARS-CoV-2 as each of these have their advantages and limitations, rendering it difficult to identify a single cell line as a definitive standard [2, 7, 8].

In this study, we analyzed the transcriptome and proteome data of hundreds of human cancer cell lines from the cancer cell line encyclopedia (CCLE) initiative [9, 10], examining for expression of SARS-CoV-2 entry mediators, ACE2 and TMPRSS2, and identified IGROV-1 as a candidate adherent cell line capable of robustly supporting the virus infection. IGROV-1 is a human ovarian carcinoma cell line and has been widely used in cancer drug-resistance studies [11]. Notably, the ovary is one of the human organs naturally expressing ACE2 [12], and primary ovarian tissue cultures have demonstrated susceptibility to SARS-CoV-2 [13]. Furthermore, ovarian function is reported to be negatively impacted in COVID-19 patients [14]. We assessed the suitability of IGROV-1 cells as a tool for SARS-CoV-2 research, as well as another human betacoronavirus HCoV-OC43, a causative agent of the common cold. Additionally, IGROV-1 cells were compared with other Vero cell lines for the isolation of SARS-CoV-2 variants from patient diagnostic samples. To demonstrate the potential applicability of using IGROV-1 cells as a tool for investigating the biology of betacoronaviruses, we conducted a transcriptome profiling of SARS-CoV-2 infected IGROV-1 cells and compared it with Huh7, a commonly used cell line for SARS-CoV-2 research, as well as primary human nasal epithelial cells (NECs). SARS-CoV-2 infection elicited a robust interferon response in IGROV-1 cells, recapitulating the response generated in primary NEC, suggesting the cell line has a non-compromised innate immune system. Furthermore, by performing genome-scale CRISPR knockout (KO) screens in IGROV-1 cells we identified Aryl hydrocarbon receptor (AHR) as a pro-viral host factor of both SARS-CoV-2 and HCoV-OC43. Finally, we performed chemical perturbation of AHR using a small molecule modulator, DiMNF (3’,4'-dimethoxy-α-naphthoflavone), to determine the role of AHR in viral production. We found that DiMNF-mediated antagonism of AHR partially altered the basal level of cellular innate immunity, consistent with a selective role of DiMNF in blocking HCoV-OC43 infection, but not SARS-CoV-2.

## Materials & methods

### Cells and viruses

African green monkey kidney Vero clone E6 (Vero E6) cells (CRL-1586, ATCC), human ovarian cancer IGROV-1 cells (the DCTD Tumour Repository, the National Cancer Institute), Rhabdomyosarcoma (RD) cells (CCL-136, ATCC), Human hepatocellular carcinoma Huh7 cells (provided by Lin-Fa Wang lab) and its Huh7.5.1 subclones (gift from Jan E Carette lab), human embryonic kidney 293T cells (CRL-3216, ATCC) and its 293FT subclones (R70007, Thermo Fisher), all were maintained in Dulbecco’s modified Eagle medium (DMEM) supplemented with 1× penicillin-streptomycin, 1× L-glutamine, and 10% heat-inactivated fetal bovine serum. NHBE cells (CC-2540, Lonza) were maintained in BEGM BulletKit Growth Media (CC-3170, Lonza).

SARS-CoV-2 isolate BetaCoV/Singapore/2/2020 (GISAID accession code EPI_ISL_406973) was used for the entire study unless otherwise stated, and was propagated as previously reported [15]. SARS-CoV-2 ancestral (SARS-CoV-2/Australia/Vic/01/20) and Omicron BA2 (hCoV-19/Australia/VIC35864/2022) were used for the DiMNF drug treatment experiments, and were propagated in Vero E6-TMPRSS2 cells cultured with DMEM supplemented with 5% fetal bovine serum (FBS) and 1% penicillin/streptomycin at 37°C, 5% CO2. Cell culture supernatants were harvested, centrifuged and aliquoted once cytopathic effect (CPE) was observed. HCoV-OC43 (VR-1558, ATCC) was propagated on RD cells. In brief, 60% confluent cells were inoculated with a multiplicity of infection (MOI) of 0.001 in 10 ml medium supplemented with 5% serum and kept at 37°C for 3 h before replenishing with fresh medium. Cells were monitored daily under the light microscope until marked CPE was visible, at 5 days post-inoculation. Virus stocks were then harvested by centrifuging the supernatant for 10 min at 500 × g to remove cellular debris, and aliquots were stored at −80°C.

### Plaque assay

Vero E6 and IGROV-1 cells were seeded on a 24-well plate at a density of 100,000 cells/well. The cells were then infected with serial-diluted SARS-CoV-2 and incubated for 1 h at 37°C. The inoculum was removed and the infected cells were replenished with 500 µl of plaque media (DMEM supplemented with 2% FBS, 0.5% Avicel and 0.5% carboxymethylcellulose). At 72 hours post infection (hpi), the plaque medium was removed the cells were fixed and stained with 4% formaldehyde and 0.5% crystal violet.

### Quantification of virus infectivity with TCID_50_

To determine the titre of HCoV-OC43, 5,000 RD cells per well were seeded in 96-well plates, in 100 µl medium supplemented with 5% FBS. Cells were then inoculated with 100 µl of a 5-fold serial dilution of the virus stock and incubated at 37°C. At 5 days post-infection, cytopathic effect was evaluated using light microscope and TCID_50_ calculated according to Spearman-Kärber method [16]. For SARS-CoV-2, 10,000 Vero E6-TMPRSS2 cells per well were seeded in 96-well plates and were utilized for titration assays. Cells were infected with 100 µl of diluted sample in quadruplicate. Cells were incubated at 37°C and 5% CO_2_ for 5 days. TCID_50_/ml titres were calculated similar to HCoV-OC43.

### Plaque reduction neutralization test (PRNT)

All monoclonal antibodies (5B7D7, AR6959, 9A9C9, AR6949, 9B1E8, 2B50, 4A1D10, AR6948) were obtained from Genscript. Serial diluted monoclonal antibodies and convalescent plasma from SARS-CoV-2 infected patients were pre-incubated with 100 PFU of SARS-CoV-2 at 37°C for 1 h. At 1 h post-incubation, the virus-antibody mixtures were added into wells pre-seeded with IGROV-1 or Vero E6 and incubated for 1 h at 37°C. After incubation, the inoculum was removed and replenished with plaque medium. At 72 hpi, the plaque media was removed and cells were fixed with 4% formaldehyde (w/v) and stained with 0.5% crystal violet (w/v).

### Pseudovirus packaging and luciferase assay

SARS-CoV-2 Wuhan-hu-1 (ancestral) and SARS-CoV BJ01 full-length spike-pseudotyped viruses were produced and packaged as previously described [17]. Briefly, 5 million 293T cells were transfected with 20 µg of pCAGGS spike plasmid using FuGene6 (Promega). At 24 h post transfection, cells were incubated with VSVΔG luc seed virus (MOI of 5) for 2 h. Following two PBS washes, infected cells were replenished with complete growth media supplemented with 1:5,000 diluted anti-VSV-G mAb (Clone 8GF11, Kerafast). At 24 h post infection, pseudoviruses were collected by centrifugation at 2,000×*g* for 5 min and kept at −80°C. The relative light unit (RLU) of the SARS-CoV-2 spike pseudotyped infected cells were determined by addition of ONE-GLO luciferase substrate (Promega) and measured using Cytation-5 microplate reader (Bio-Tek).

### Microscopy and immunofluorescent imaging

SARS-CoV-2 immunofluorescence assay was performed in a biosafety level 3 laboratory. Briefly, SARS-CoV-2-infected IGROV-1 and Vero E6 cells were fixed with 4% formaldehyde at 48 hpi (MOI of 0.1) for 30 min. The SARS-CoV-2 antigens were immunostained with human anti-SARS-CoV-2 spike monoclonal antibody AR6959 for 30 min at 37°C, followed by PE-label anti-human IgG polyclonal antibody (eBioscience) for 30 min at 37°C. Nuclei were stained with DAPI for 10 min at room temperature. The fluorescence images were captured at 40× using an epifluorescence microscope.

For the images of HCoV-OC43 CPE induction in IGROV-1 cells, monolayers of 1 × 10^5^ cells were grown in 24-well plates for approximately 16 h, then infected with HCoV-OC43 at three different MOIs (MOI 0.1, 1, and 10) in DMEM supplemented with 2% FCS, 1× penicillin-streptomycin, and 1× L-glutamine. Virus was adsorbed for 3 h, then virus inoculum was removed, and fresh growth media were added. After infection, cell morphology was monitored daily, and CPE was observed using a brightfield microscopy. The brightfield images were taken at 96 hpi.

### Lentiviral packaging

Lentiviruses were packaged by the 3rd generation lentivirus packaging system plasmids pRSV-Rev, pMD2 VSV-G, and pMDLg/pRRE as previously described [18] with modifications. In brief, 293FT cells were seeded and cultured at 37°C for 16–24 h. At 2 h prior to transfection, the cells were replenished with IMDM medium (Gibco Life Technologies) containing 1× penicillin-streptomycin, 1× L-glutamine, and 10% heat-inactivated fetal bovine serum. Cells were then co-transfected with all three plasmids in addition to the human CRISPR Brunello library (Addgene, #73178) using TransIT-LT1 Transfection Reagent (Mirus Bio LLC). Supernatants containing the lentiviral particles were harvested at 48 and 72 h post-transfection, aliquoted, and stored at −80°C.

Lentiviral transduction was performed on AHR KO cell lines to generate cells that stably express the proteins of interest. Constructs to express AHR cDNA or GFP were cloned by Gibson Assembly (New England Biolabs) into a lentiCas9-Blast backbone (Addgene, #52962), replacing the Cas9 protein. Lentiviruses were produced by co-transfection of the transgene expressing plasmid with a mixture of pRSV-Rev, pMD2 VSV-G, pMDLg/pRRE, and pAdVAntage packaging plasmids into 293FT cells using FuGENE HD (Promega). At 48 h post-transfection, lentivirus was harvested from the supernatant and filtered through a 0.45-micron filter. 1× protamine sulphate was added to the lentivirus before transducing respective cell lines overnight. IGROV-1 cells stably expressing the protein of interest were selected by treatment with 15 μg/ml of blasticidin along with untransduced cells as negative controls.

### Generation of isogenic KO cells

Single-cell derived AHR KO clone was generated using a CRISPR/Cas9 editing strategy. Firstly, single guide RNA (sgRNA) sequence against AHR was obtained from human CRISPR Brunello lentiviral pooled libraries (Addgene, #73178). DNA oligo containing sgRNA sequence 5’-TACCACATCCACTCTAAGCA-3’ was annealed and ligated into the Cas9-expressing pX458 plasmid (Addgene, #48138) generated by the Zhang lab. IGROV-1 cells were transfected with the pX458 constructs using TransIT-LT1 transfection reagent (Mirus Bio LLC) according to the manufacturer’s protocol. 48 h post-transfection, GFP positive cells were single-cell sorted into 96-well plates using a BD Influx cell sorter at the Duke-NUS FACS facility. Colonies were expanded from single cells. For genotyping, genomic DNA was isolated from the expanded clones and the sgRNA-targeted site was PCR amplified using primers pair of 5’-TGTCCCTTTCTGAATTCAATTACA-3’ and 5’-TTGAAAGAGCCCTGGGTGAC-3’. The sequence of the PCR product was determined by Sanger sequencing. The isogenic KO cells verified by genotyping were further confirmed by Western blotting.

### Genome-scale CRISPR/Cas9 knockout screens

IGROV-1 cells were stably transduced with lentiCas9-Blast (Addgene, #52962) and subsequently selected using blasticidin (15 μg/ml) to generate a population of Cas9 expressing IGROV-1 cells, i.e. IGROV-1-Cas9. These cells were subsequently transduced with the Human CRISPR Knockout Pooled Library (Brunello) (Addgene, #73178) at MOI of 0.3. Cells were selected using puromycin (1.5 μg/ml) and heterogeneous knockout cell populations were subsequently pooled together. The CRISPR genetic screens were started 10 days post transduction. Cell libraries displaying a >700-fold coverage of mutagenized cells were infected with SARS-CoV-2 at the Duke-NUS ABSL3 facility at a MOI of 0.1, and HCoV-OC43 in a Duke-NUS BSL2 laboratory at a MOI of 0.05.

SARS-CoV-2 infection was repeated for an additional round at two weeks post-infection. A collection of virus-resistant cell colonies were pooled and harvested at one month post-infection. HCoV-OC43 infection was also repeated for two additional rounds at 5 and 10 days post-infection before virus-resistant cell colonies were pooled and harvested at 24 days post-infection. A total number of 40 million uninfected starting populations of mutagenized cells were used as the unselected control. Total genomic DNA from both virus-resistant and uninfected control cells was extracted using QIAamp DNA Mini Kit (Qiagen). The inserted guide RNA sequences were retrieved from the genomic DNA by PCR amplification. The PCR products were then purified and subjected to HiSeq platform (Illumina) next-generation sequencing. The sequencing data were processed and analyzed using MAGeCK (v0.5.9) [19] to determine the ranking of each hit by taking the following criteria into account: the number of sequencing reads for each unique guide, the number of selected guide RNA per gene, and the enrichment of a particular guide RNA in comparison to uninfected cell populations. More than 97% of the total unique gRNAs in the library used (human Brunello comprises approximately 80,000 individual plasmids) were recovered in the unselected sample, demonstrating successful transduction of the WT IGROV-1 cells with the lentivirus pool which paved the way for the subsequent analysis of the virus selected samples.

### Western blotting analysis

Cells were lysed in RIPA buffer (Thermo Fisher). Cell lysates were denatured in the presence of 4× Laemmli sample buffer (Bio-Rad) containing 5% beta-mercaptoethanol at 95°C for 10 min. Samples were separated by SDS-PAGE on pre-cast 4-15% poly-acrylamide gels (Bio-Rad) using Mini-PROTEAN gel system and transferred onto PVDF membranes (Bio-Rad). The PVDF membranes were blocked with 5% blotting grade blocker (Bio-Rad) dissolved in 1× phosphate buffered saline (Corning) containing 0.1% Tween-20 (Sigma). Subsequently, membranes were incubated at 4°C overnight with a primary antibody diluted in the blocking buffer. The following primary antibodies were used to detect the presence of indicated proteins in this study: GAPDH (GeneTex, GTX627408, 1:5,000), TMPRSS2 (Santa Cruz, sc-515727, 1:1,000), AHR (Proteintech, 17840-1-AP, 1:1,000), and HCoV-OC43 Nucleocapsid (Merck, MAB9012, 1:3,000). After washing three times using PBS 0.1% Tween-20, membranes were incubated with secondary antibodies coupled to HRP (Genetex, GTX213111-01 (anti-mouse) or GTX213110-01 (anti-rabbit), 1:10,000) for 1 h at room temperature. Membranes were then washed three times before they were subjected to SuperSignal West Pico PLUS Chemiluminescent Substrate or Dura Extended Duration Substrate (Thermo Fisher) peroxide solutions treatment for visualization of antibody-bound proteins on ChemiDoc Touch imaging system (Bio-Rad).

### Analysis of CCLE data for ACE2 and TMPRSS2 expression

Normalized transcriptome data of all cell lines available was downloaded from CCLE database website (https://sites.broadinstitute.org/ccle/) (update 2018-09-29). Proteome data was obtained from Nusinow *et al*. [10]. The subset of ACE2 and TMPRSS2 expression values from the datasets are available in (Sup Table 1).

### SARS-CoV-2 isolation from patients samples

A 20 µl aliquot of a patient nasal swab sample was added to one well of a 24-well plate containing either Vero E6, Vero E6-TMPRSS2, Vero E6-TMPRSS2-ACE2, Vero E6-SLAM or IGROV-1 cells. Supernatant was collected for qPCR one hour after inoculation (P0) and 1 h after the passage (P1). Nucleic acid was extracted using a QIAamp Viral RNA Mini Kit (Qiagen) according to manufacturer’s instructions. In brief, a 140 μl volume of each sample was used for extraction and eluted in 60 μl of elution buffer. Reverse transcription of viral RNA to complimentary DNA (cDNA) was performed using Bioline SensiFAST cDNA synthesis kit (Bioline Reagents Ltd) according to manufacturer’s instruction. In brief, a 10 μl volume of eluted nucleic acid was added to 10 μl of SensiFAST cDNA reaction mix, heat linearized at 25°C for 10 min then incubated at 42°C for 15 min before denaturation at 85°C for 5 min on an Applied 50 Biosystems SimpliAmp thermal cycler (Thermo Fisher Scientific). RT-PCR was performed as previously described [20] with the 5’-CACATTGGCACCCGCAATC-3’ (forward) and 5’-GAGGAACGAGAAGAGGCTTG-3’ (reverse) primers, and FAM-ACTTCCTCAAGGAACAACATTGCCA-BBQ probe was used for the quantification.

### Growth kinetics measurement of HCoV-OC43 by RT-qPCR

1 × 10^5^ IGROV-1 cells were grown to approximately 80% confluency in 24-well plate and inoculated with 0.5 ml HCoV-OC43 inoculum at MOI of 0.1, 1, or 10. After incubation at 37°C for 3 h, the inoculum was removed, and 1 ml fresh medium was added for further incubation. Cells were harvested at 0 and 6 days post-infection and total RNA was extracted from 150 ml of the supernatant using the Direct-zol RNA MiniPrep kit (Zymo) according to manufacturer’s instruction. qRT-PCR was performed with 5 ml of extracted total RNA using AgPath-ID One-Step RT-PCR Reagents (Applied Biosystems) with 5’-CGATGAGGCTATTCCGACTAGGT-3’ (forward) and 5’-CCTTCCTGAGCCTTCAATATAGTAACC-3’ (reverse) primers and FAM-TCCGCCTGGCACGGTACTCCCT-BHQ1 probe. HCoV-OC43 Nucleocapsid sequences were cloned into pGEM-T vector (Promega), and cycle threshold values were converted to viral genome copies per microliter based on regression analysis of plasmid dilutions.

### RNAseq analysis

For RNA sequencing analysis, NHBE cells were seeded in 6-well plate (3 × 10^5^ cells per well) and were incubated for 16 h. Prior to infection, the cells were pre-treated with 2 ml medium containing 1 μM of DiMNF or 0.003% DMSO for 48 h. NHBE cells were then inoculated with 2 ml of HCoV-OC43 inoculum (MOI 5) and incubated for 3 h. Subsequently, the inoculum was removed from the cells and 2 ml fresh medium containing 1 μM of DiMNF or 0.003% DMSO was added. At 48 h post-infection, total RNA was extracted using RNeasy Mini Kit (Qiagen), as per manufacturer’s instructions. Next generation sequencing was done using an illumina HiSeq machine followed by demultiplexing. The quality of raw fastq files were checked with FastQC (v0.11.9, https://www.bioinformatics.babraham.ac.uk/projects/fastqc/), and rRNA reads were removed using BBDuk (From BBTools v38.98, https://sourceforge.net/projects/bbmap/). Trimmed fastq files were aligned to human reference genome hg38 (release 13) or HCoV-OC43 genome (https://www.atcc.org/products/vr-1558) using STAR aligner (v2.7.10a) [21]. Gene count tables were generated from bam files using featureCounts from subread software (v2.0.3) [22], before subjecting to analysis with edgeR (v3.40.2) [23] in R to determine differential gene expression (DGE). Innate immunity genes were obtained from InnateDB database and manual addition [24]. Gene Ontology over-representation analysis for biological pathways, as well as KEGG pathway enrichments were done using clusterProfiler package (v4.8.1) [25] in R.

Time-series RNASeq samples of IGROV-1 and Huh7 cells infected with SARS-CoV-2 were analysed similarly. The NEC raw fastq files were obtained from GEO under accession number GSE162131. Gene set enrichment analysis (GSEA) was performed pre-ranked, using the product of -Log(*P*-values) and Log(fold-change), and calculated using the fgsea package (v1.16.0) with the parameters minSize = 15, maxSize = 500 and nPermSimple = 100000. Heatmap was generated using the pheatmap package (v.1.0.12, https://cran.r-project.org/web/packages/pheatmap/) and the gene counts used to generate the heatmap were log normalised using rlog from DESeq2 package (v1.30.1) [26].

### Antiviral experiments

DiMNF (SML0079, Sigma-Aldrich) was resuspended in DMSO (Sigma-Aldrich). IGROV-1 or NHBE cells were seeded on 96-well plate at a seeding density of 7,000 cells/well. IGROV-1 cells were treated with DiMNF at concentrations ranging from 0.094 μM to 1.5 μM, and NHBE cells were treated with DiMNF at 0.5, 0.75, and 1 μM. IGROV-1 cells were inoculated with HCoV-OC43 at MOI 0.5; and NHBE cells were inoculated with HCoV-OC43 at MOI 5. At 72 hpi, viral production by infected cells was measured using TCID50 assay. Medium containing 1 μM (0.01%) and 1.5 μM (0.015%) DMSO were used as controls without DiMNF.

Remdesivir was obtained from TargetMol and chloroquine diphosphate was obtained from Sigma. Approximately 10,000 cells in a 96-well plate were infected with SARS-CoV-2 at a MOI of 0.1 for 1 h in the presence of increasing concentration of the drug. At 1 hpi, the inoculum was removed and the cells were replenished with the media containing 2% FBS and increasing concentration of the targeted drug.

### Cell viability assay

IGROV-1 or NHBE cells were seeded on 96-well plate at a seeding density of 7000 cells/well. IGROV-1 cells were treated with DiMNF at concentrations ranging from 0.094 μM to 1.5 μM, and NHBE cells were treated with DiMNF at 0.5, 0.75, and 1 μM. Medium containing 1 μM (0.01%) and 1.5 μM (0.015%) of DMSO were used as control without DiMNF. At 72 h post-treatment, the viability of IGROV-1 or NHBE cells treated with DiMNF or DMSO was measured by cell viability assay using CellTiter-Glo reagent (G9241, Promega) according to manufacturer’s instructions. Briefly, spent growth medium was aspirated and replaced with 50 ml CellTiter-Glo reagent. The sample was mixed gently and transferred to a white 96-well flat bottom plate (3912, Costar) and luminescence was measured in a Tecan Infinite M200 plate reader.

### Statistical analysis

GraphPad Prism 9 (GraphPad Software) was used to analyse and visualize the data.

## Results

### IGROV-1 cell line is susceptible and permissive to SARS-CoV-2 and HCoV-OC43

**Figure 1.**
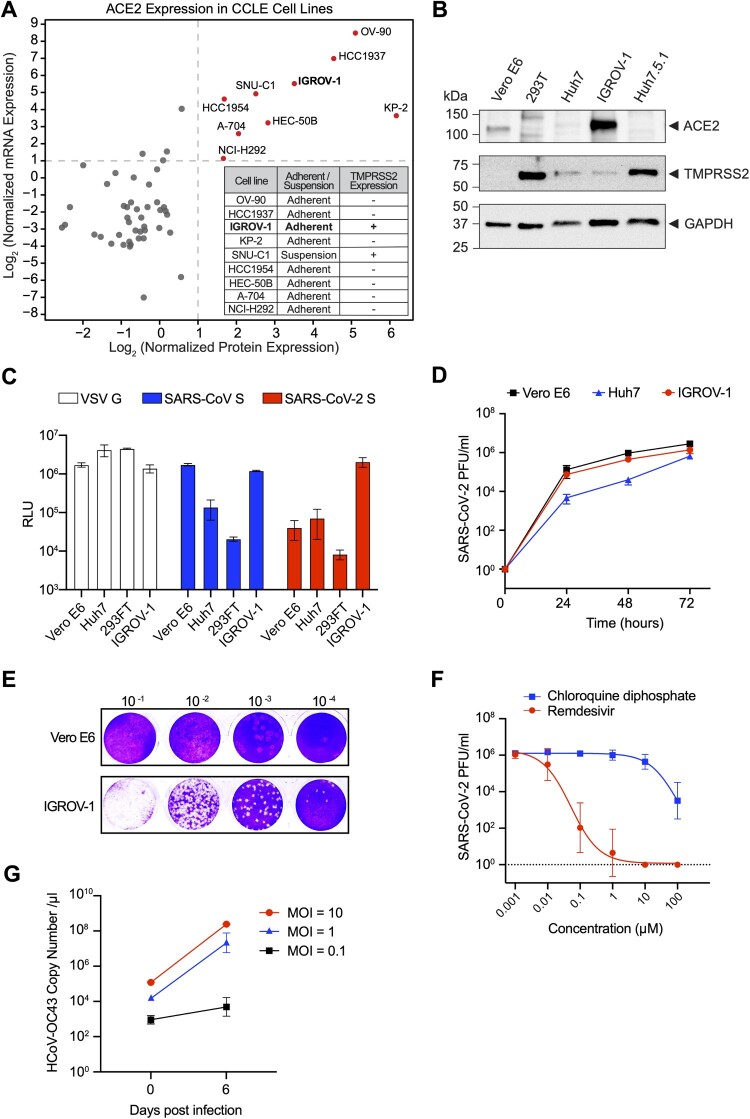
Identification and characterization of IGROV-1 as a susceptible and permissive cell line for SARS-CoV-2 and HCoV-OC43. (A) ACE2 mRNA and protein expression levels in CCLE database (scatter plot), as well as TMPRSS2 protein expression (table). Normalized expression values are used for the plot with cutoffs at log_2_ expression value of 1. (B) ACE2 and TMPRSS2 protein levels in IGROV-1, Vero E6, 293T, Huh7 and Huh7.5.1 cell lines. GAPDH levels were used as internal controls. (C) Renilla luciferase signal measurement in IGROV-1, Vero E6, 293FT, and Huh7 cells, after infection with viruses pseudotyped with SARS-CoV-2 S, SARS-CoV S and VSV G proteins. Data are represented as mean ± SEM (n = 2). (D) SARS-CoV-2 replication kinetics in IGROV-1, Huh7 and Vero E6 cells over 72 h, quantified by plaque assay. Data are represented as mean ± SEM (*n* = 3). (E) Representative plaque assay plates using IGROV-1 or Vero E6 cells to measure SARS-CoV-2 viral titre. Both plates were subjected to the same set of virus inoculums, with 10 to 10,000 fold dilution. (F) SARS-CoV-2 viral inhibition in IGROV-1 cells, upon treatment with a gradient concentration of chloroquine diphosphate or remdesivir. Viral titres were quantified by plaque assay. Data are represented as mean ± SEM (*n* = 4). (G) HCoV-OC43 propagation kinetics in IGROV-1 cells. Three different MOIs (0.1, 1, 10) were used and genome copies were quantified by real time qPCR. Data are represented as mean ± SEM (*n* = 3).

All viruses must hijack specific host factors to enter the cells. In the case of SARS-CoV-2, the spike protein (S) first binds to the cellular receptor, ACE2, then TMPRSS2-mediated proteolytic trimming enables the virus to fuse at the cell plasma membrane. This results in deposition of the viral genome into the cytoplasm, and is the preferred entry route of SARS-CoV-2 in human airway and lung cells [6]. To identify human cell lines that support SARS-CoV-2 infection, we first analyzed the CCLE cancer cell line database to select candidate cell lines with the highest levels of ACE2 mRNA and protein expression. A total of nine cell lines were shortlisted (OV-90, HCC1937, IGROV-1, KP-2, SNU-C1, HCC1954, HEC-50B, NCI-H292 and A-704), which were further evaluated for TMPRSS2 protein expression (Figure 1(A)). Only two of the nine cell lines, IGROV-1 and SNU-C1, were found to express detectable levels of TMPRSS2 protein. As SNU-C1 is a suspension cell line, we focused on characterizing the potential utilization of IGROV-1 cell line for SARS-CoV-2 research. We first confirmed ACE2 and TMPRSS2 expression in IGROV-1 cells using immunoblotting (Figure 1(B)). The protein level of ACE2 exceeded all other cell lines, and the level of TMPRSS2 was comparable to Huh7 cells. To evaluate susceptibility of IGROV-1 cells to infection, we used non-replicating luciferase-expressing reporter viruses pseudotyped with the S protein of the SARS-CoV and SARS-CoV-2 (Figure 1(C)). A VSV glycoprotein (G) pseudotyped virus was used as a positive control for pseudovirus entry. We observed that IGROV-1 was highly susceptible to both SARS-CoV and SARS-CoV-2 S pseudotyped reporter viruses, as compared to Vero E6, Huh7 and 293FT cell lines. Next, we measured the replication kinetics of SARS-CoV-2 in IGROV-1 cells to assess permissibility. SARS-CoV-2 displayed identical replication kinetics over 72 h in both IGROV-1 and Vero E6, and these viral titres were higher than those obtained in Huh7 cells (Figure 1(D)). Similarly, comparable levels of SARS-CoV-2 S were observed in IGROV-1 and Vero E6 cells infected with the virus (Sup Figure 1(A)). Notably, IGROV-1 cells depict pronounced CPE following SARS-CoV-2 infection, rendering them an appropriate cell line to be used in assays measuring viral titre based on CPE (Figure 1(E) and Sup Figure 1(B)). Vero cells do not express TMPRSS2 and SARS-CoV-2 primarily depends on the endosomal membrane fusion pathway to infect these cells, thus inhibitors of endosomal trafficking and acidification can biasedly inhibit SARS-CoV-2 infection in Vero cells [27]. However, such inhibitors were found ineffective to prevent SARS-CoV-2 infection in human airway cells as the membrane fusion can occur at both the plasma membrane and endosomes [5]. Expectedly, we observed that SARS-CoV-2 infection in IGROV-1 cells can be efficiently inhibited by remdesivir, a nucleoside analog drug, but not chloroquine, an endosomal acidification inhibitor (Figure 1(F)), demonstrating viral entry in IGROV-1 cells recapitulates entry in human airway cells. We further evaluated whether another human betacoronavirus, HCoV-OC43, could efficiently infect IGROV-1 cells. We observed robust replication of HCoV-OC43 viral genome in IGROV-1 cells, and obvious CPE following infection (Figure 1(G) and Sup Figure 1(C)).

### Utilizing IGROV-1 for SARS-CoV-2 serological assays and assessing virus isolation from patient samples

**Figure 2.**
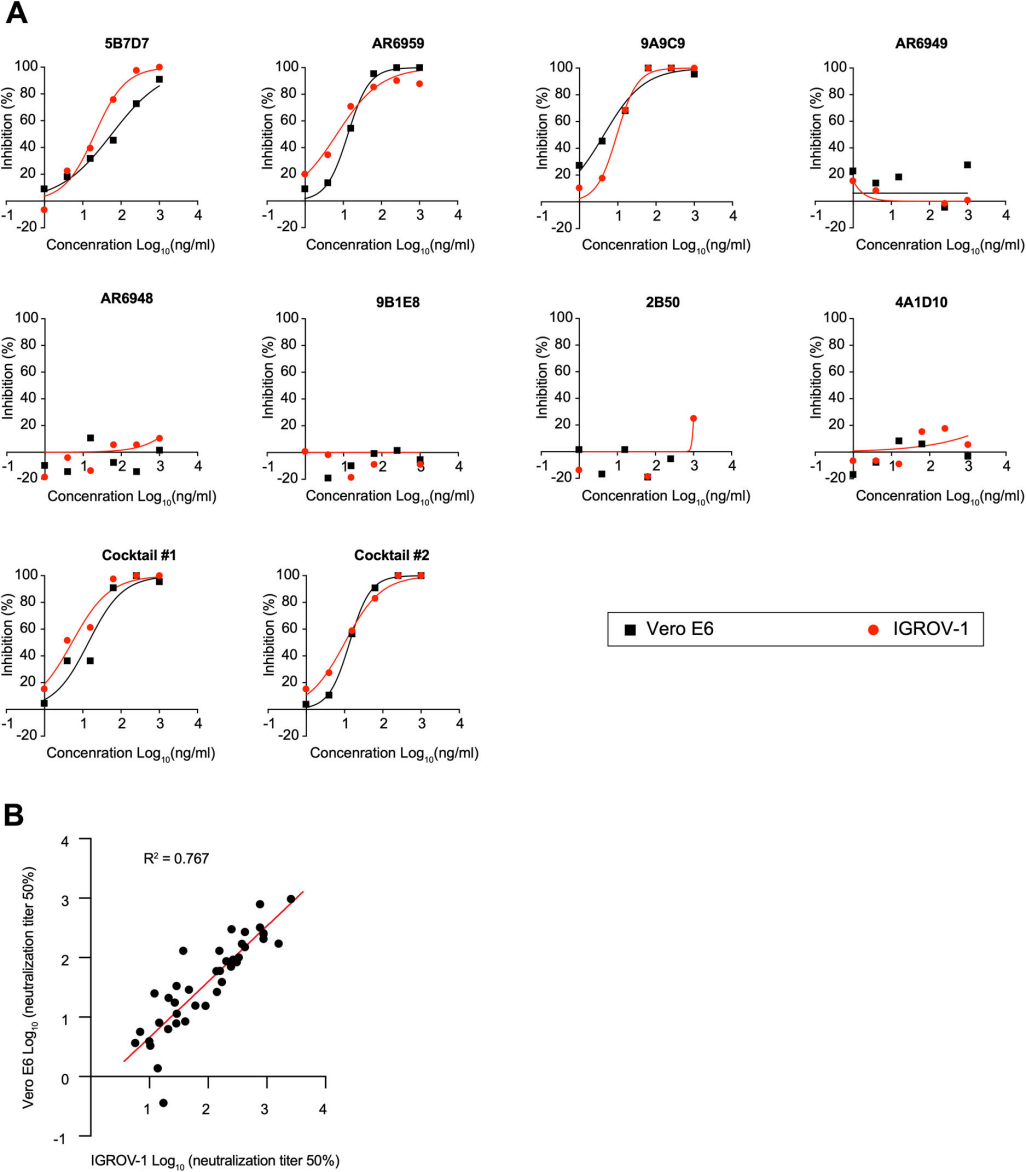
Application of IGROV-1 for serological neutralization assays. (A) Determining the neutralization potency of eight commercial SARS-CoV-2 monoclonal antibodies to inhibit viral infection in IGROV-1 and Vero E6 cells, measured by PRNT_50_ assay. Monoclonal antibody cocktail #1: AR6949 and AR6959. Cocktail #2: 9A9C9, AR6949 and AR6959. (B) Paired PRNT_50_ values for convalescent sera tested on IGROV-1 and Vero E6 cells. Correlation is determined by Pearson method.

We performed plaque reduction neutralization tests (PRNT) in IGROV-1 cells using a panel of monoclonal antibodies with different neutralization activities against SARS-CoV-2. Overall, the yielded neutralization activity of the antibodies was comparable in SARS-CoV-2 infected IGROV-1 and Vero E6 (Figure 2(A)). Using sera derived from COVID-19 patients, we also demonstrated that neutralization titres obtained using IGROV-1 cells were strongly correlated with those obtained using Vero E6 cells (Figure 2(B)).

To determine whether IGROV-1 cells are suitable for virus isolation from clinical samples, we selected five patient nasal swab samples where virus isolation had been previously attempted. IGROV-1 and four Vero cell line derivatives were inoculated with patient nasal swabs and incubated for 7 days, or less if CPE was evident. Supernatant from each sample (P0) was frozen and passaged once (P1). RNA was extracted from P0 and P1 supernatants and viral RNA was measured by qPCR (Table 1). In the four samples where virus isolation was previously successful, replication in IGROV-1 cells was efficient, as measured by a decrease in qPCR Ct value from P0 to P1. In one sample (Patient 3, SARS-CoV-2 isolate BA.2.12.1) the virus could only be propagated in IGROV-1 cells, among all other cell lines tested. Virus replication did not occur in any cell line inoculated with the sample from patient 5, where previous isolation was unsuccessful. These results demonstrate that IGROV-1 cells are equally efficient to Vero cell lines and provide better consistency for isolating virus from patient samples.

**Table 1.** Isolation of SARS-CoV-2 from patient samples in multiple cell lines, measured by qPCR.

Patient number	Previously isolated	GISAID ID	Strain	Passage	Cell line				
					Vero E6	Vero E6 – TMPRSS2	Vero E6 – TMPRSS2-ACE2	Vero E6 – SLAM	IGROV-1
1	Yes	NA	NA	P0	27.43	27.87	27.86	27.30	27.77
				P1	43.65	15.52	17.05	22.58	25.91
2	Yes	EPI_ISL_962831	B.1.1.7	P0	29.72	29.52	30.34	31.01	29.97
				P1	neg	15.84	17.63	23.66	17.64
3	Yes	EPI_ISL_12391374	BA.2.12.1	P0	26.04	27.79	28.35	31.80	32.00
				P1	neg	44.20	neg	neg	16.13
4	Yes	EPI_ISL_2920929.2	B.1.351	P0	26.55	27.77	26.79	27.35	27.38
				P1	18.79	15.71	15.83	18.01	17.14
5	No	EPI_ISL_1913207	B.1.617.2	P0	neg	neg	neg	neg	neg
				P1	neg	neg	neg	neg	neg

Numbers in cell line columns are qPCR Ct values. Ct > 45 is denoted as “neg.”

### Transcriptomic profiling of SARS-CoV-2 infected IGROV-1 cells

**Figure 3.**
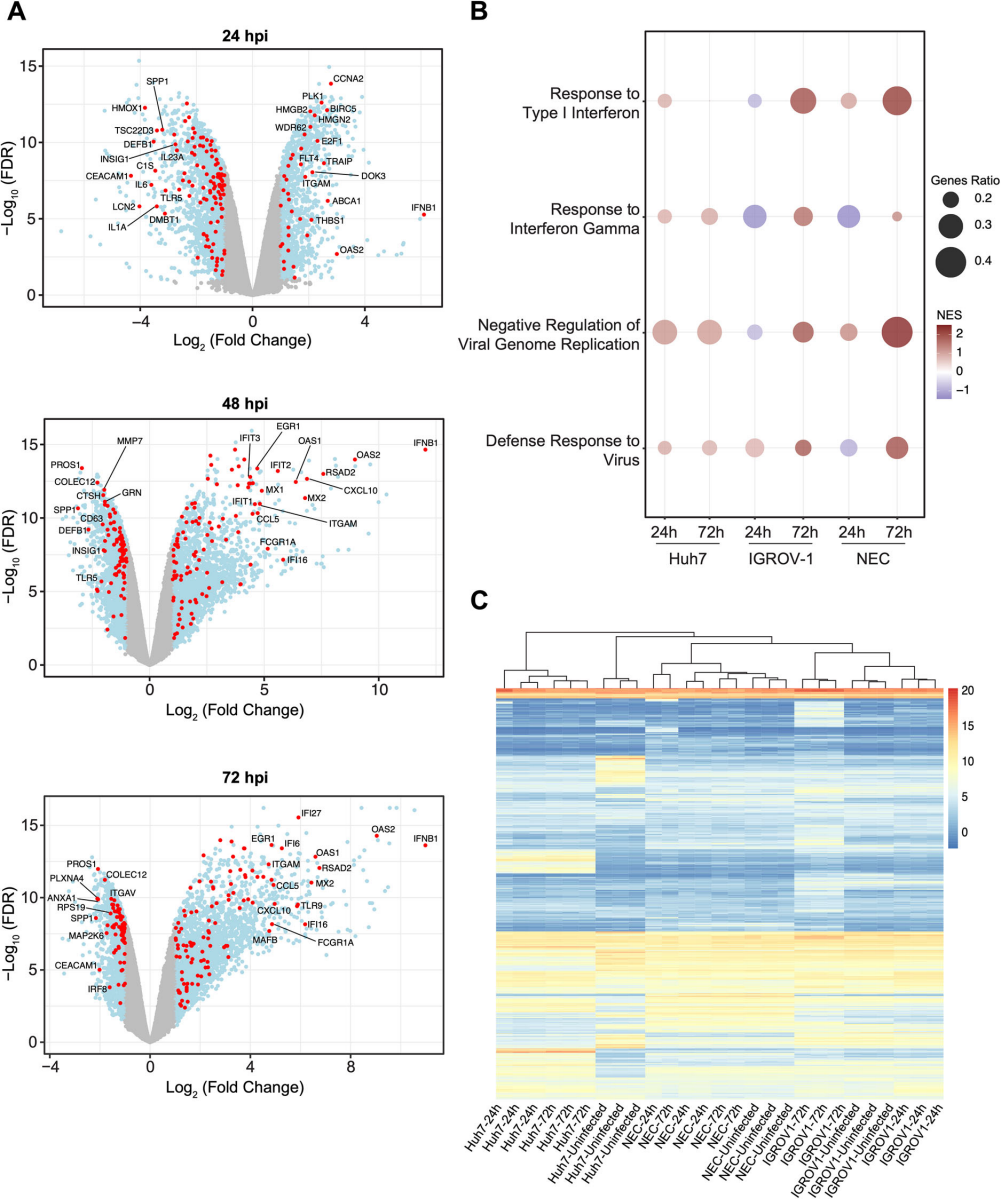
Transcriptional profiling of SARS-CoV-2 infection in IGROV-1 cells. (A) Differentially expressed genes (light blue) and innate immunity genes (red) in IGROV-1 cells at 24, 48 and 72 hpi, compared to 0 (no virus control). (B) Key antiviral pathways as functionally annotated using Gene Set Enrichment Analysis (GSEA). Size of bubble refers to proportion of genes from gene set lying at leading edge. Colours indicate Normalised Enrichment Scores (NES). Only significant pathways are plotted. (C) Heatmap of log normalised counts from all three cell types and both time-points, clustered by genes and libraries. Genes are selected as follows: top 200 differentially expressed genes by fold change for each condition (cell type, hpi) are selected, and a union of gene lists across all conditions is taken, totalling 935 genes. All conditions are performed in triplicates (*n* = 3).

To further characterize IGROV-1 cells, comparative bulk RNA sequence analysis was conducted on IGROV-1 and Huh7 cells following SARS-CoV-2 infection. Total RNA was extracted at 0, 24, 48 and 72 hpi. Viral titres were previously shown to increase over time by plaque assay (Figure 1(D)). Differential gene expression analysis was performed by comparing all time points to the uninfected control, revealing that Huh7 cells exhibited a consistent number of differentially expressed genes (DEGs) across all time points (Sup Figure 2). In contrast, IGROV-1 cells demonstrated a progressive increase in the number of DEGs as a function of time (Figure 3(A)). Notably, several key components of innate immunity including IFNB1, a type 1 interferon essential for anti-viral response, and known interferon stimulated genes (ISGs) including OAS2, exhibited a marked induction at 24 hpi, followed by a further increase in induction at 48 hpi, and sustained expression at 72 hpi.

To further evaluate the suitability of utilizing IGROV-1 cells for studying betacoronaviruses, we compared our transcriptomic results with a previously published dataset studying SARS-CoV-2 infection in NECs. This study employed a similar experimental design, and focused on the 24 and 72 hpi timepoints [28]. First, we performed a gene set enrichment analysis for functional annotation of DEGs from Huh7, IGROV-1 cells and NECs, summarizing significantly enriched biological pathways (Sup Table 2). Notable similarities were observed in the induction patterns of critical antiviral pathways between IGROV-1 cells and NECs, including a gradual upregulation and a more pronounced induction of interferons. In contrast, Huh7 cells exhibited an earlier but relatively weaker response to SARS-CoV-2 infection, which remained constant and did not exhibit further induction (Figure 3(B)). These findings suggest that IGROV-1 cells can similarly replicate the antiviral transcriptional profiles observed in primary cells like NECs.

To gain a better insight into the overall transcriptional similarities among the three cell types, we employed a systematic approach analyzing the hierarchical clustering of the cells for a subset of top 200 DEGs from all cell types (based on fold-change and considering both up and downregulated genes) (Sup Table 3). As anticipated, libraries derived from the same cell type and condition were clustered closely together, indicating their intrinsic similarity (Figure 3(C)). Notably, IGROV-1 cells and NECs formed a distinct cluster when comparing cell types, while Huh7 cells displayed a dichotomy between infected and non-infected states within the same level of the hierarchy. Collectively, our transcriptional profiling demonstrated the robust antiviral response generated by IGROV-1 cells and highlighted their greater transcriptional similarity to primary cells rather than widely used Huh7 cells.

### AHR is a host dependency factor of SARS-CoV-2 and HCoV-OC43

**Figure 4.**
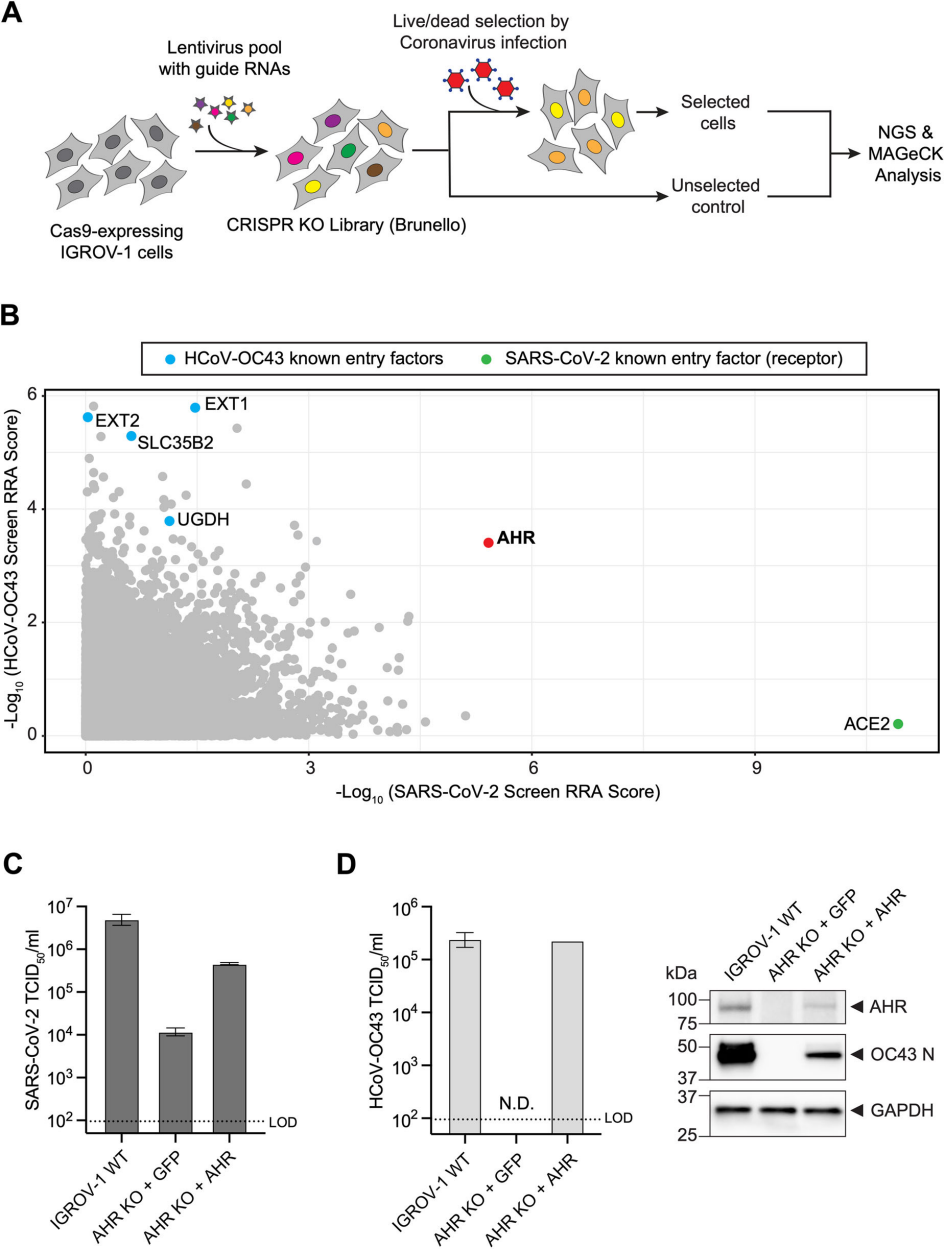
Genome-scale CRISPR screens identified AHR as a common host factor for SARS-CoV-2 and HCoV-OC43. (A) Schematic diagram of CRISPR-Cas9 KO screen workflow. (B) Overlapped MAGeCK RRA scores of SARS-CoV-2 (x axis) and HCoV-OC43 screens (y axis). The values depicted are – Log_10_ of negative selection scores of the gene summary output files. (C) SARS-CoV-2 viral titre in IGROV-1 WT, AHR KO, and AHR KO with cDNA complementation. KO cells were transduced with a lentivirus carrying GFP protein to control for unspecific lentivirus transduction effects. Data are represented as mean ± SEM (*n* = 3). (D) HCoV-OC43 viral titre in IGROV-1 WT, AHR KO, and AHR KO with cDNA complementation (left), as well as nucleocapsid (N) protein accumulation following infection (right). KO cells were transduced with a lentivirus carrying GFP protein to control for unspecific lentivirus transduction effects. TCID_50_ data are represented as mean ± SEM (*n* = 3). GAPDH levels were used as internal controls for the western blots. LOD denotes the “limit of detection.” N.D. denotes “not detected.”

Multiple groups have performed genetic screens aiming to identify SARS-CoV-2 host factors [29–34]. Despite the identification of multiple important proteins (e.g. TMEM41B, TMEM106B), there is little overlap across these studies. This can be attributed to variation in the screening conditions, such as the use of different libraries, viral strains, analysis algorithms, and importantly, the cell lines chosen for the screening experiments. Using the human Brunello library [35], we performed genome-wide CRISPR KO screens in IGROV-1 cells with both SARS-CoV-2 and HCoV-OC43, to identify cellular host factors required for human betacoronavirus infection (Figure 4(A)). The library of CRISPR mutagenized cells were infected with SARS-CoV-2 and HCoV-OC43 at a MOI of 0.1 and 0.05, respectively, and resistant cells surviving the cytolytic selections were harvested for next generation sequencing (NGS) and bioinformatic analysis. ACE2 scored as the top hit in the SARS-CoV-2 screen, and multiple components of the heparan sulphates pathway (EXT1, EXT2, SLC35B2) dominated the top hits in the HCoV-OC43 screen, validating the screening outcome as these host factors are well-defined entry mediators [29, 36]. Aryl hydrocarbon receptor (AHR) was identified as a hit in both screens, ranking 2nd for SARS-CoV-2 and top 50th for HCoV-OC43 (Figure 4(B)) (Sup Table 4).

To confirm that AHR is a host dependency factor of SARS-CoV-2 and HCoV-OC43, we generated clonal AHR KO cells. We observed that SARS-CoV-2 production was significantly reduced (∼2.5 Log) in AHR depleted cells in comparison to the wild-type (WT) IGROV-1, and this reduction was partially restored by ectopic expression of AHR cDNA in the KO cells (Figure 4(C)). Likewise, HCoV-OC43 infection was diminished in the AHR KO cells, as evidenced by both the infectious viral particle production and nucleocapsid (N) protein accumulation in the KO cells, which could also be rescued with AHR cDNA complementation (Figure 4(D)).

### DiMNF selectively inhibits HCoV-OC43 viral infection, but not SARS-CoV-2

Giovannoni *et al.* previously found AHR to be upregulated in some COVID-19 patients. They demonstrated that CH223191, an AHR inhibitor, can effectively inhibit SARS-CoV-2 and HCoV-229E infection in several mammalian cell lines [37]. Recently, Shi *et al.* have also employed CH223191 to block SARS-CoV-2 infection both *in vitro* and *in vivo* [38]. Clofazimine, another AHR inhibitor, has also been recently reported as a broad-spectrum coronavirus inhibitor *in vivo* [39, 40]. This data supports the potential of AHR inhibitors to be employed as antiviral agents.

**Figure 5.**
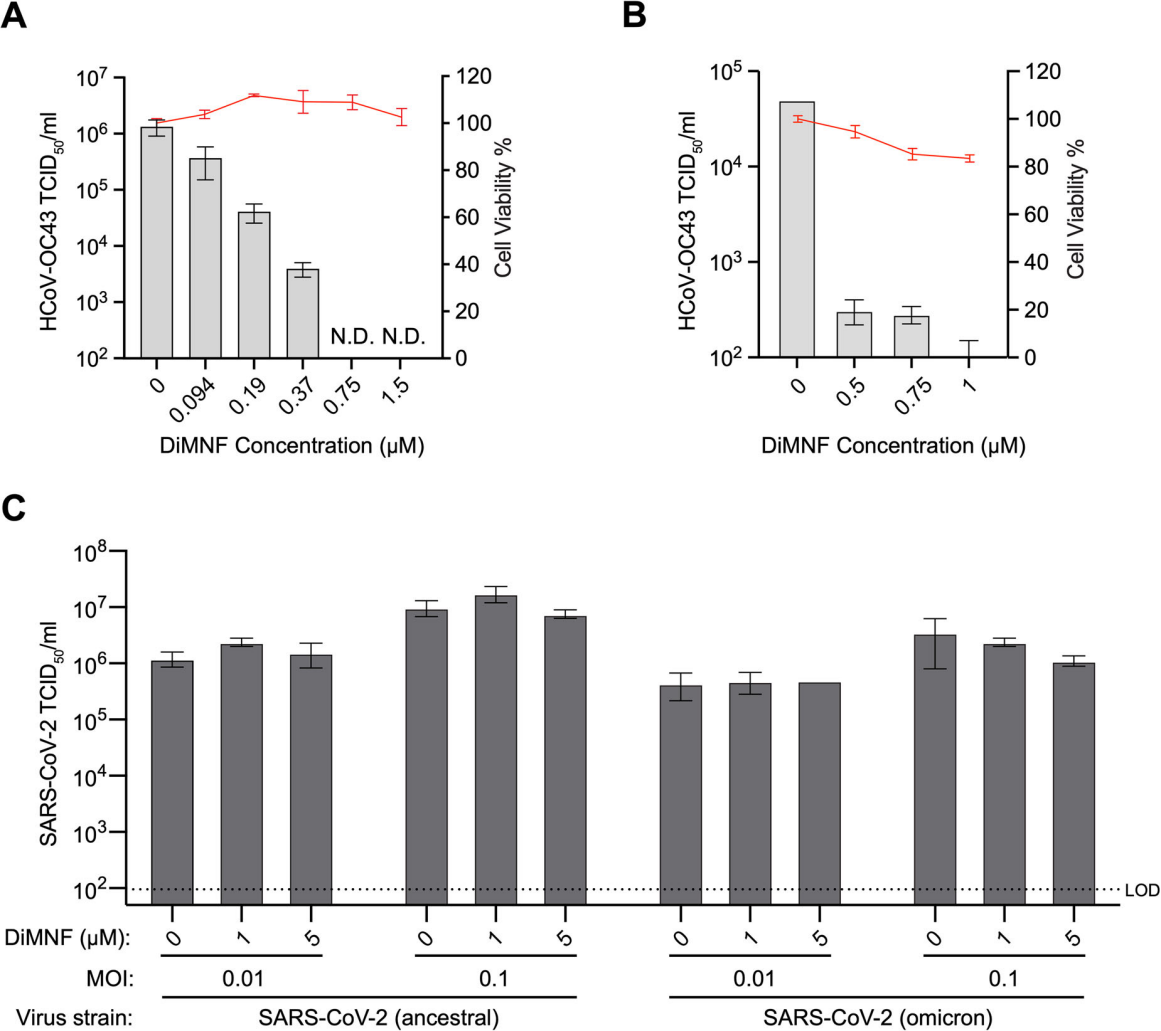
AHR modulator, DiMNF, inhibited HCoV-OC43 infection but not SARS-CoV-2. (A and B) HCoV-OC43 viral titre inhibition in response to DiMNF treatment in (A) IGROV-1 and (B) NHBE cells (*n* = 3). Cell cytotoxicity of DiMNF is measured independently from the infection experiments, via CellTiter-Glo assay (*n* = 4). Data are represented as mean ± SEM. N.D. denotes “not detected.” (C) SARS-CoV-2 viral titre could not be inhibited in response to DiMNF treatment of IGROV-1 cells. Data are represented as mean ± SEM (*n* = 3). LOD denotes the “limit of detection.”

We further assessed the antiviral properties of an additional modulator of AHR, DiMNF, against HCoV-OC43 and SARS-CoV-2. We observed a dose-dependent inhibition of HCoV-OC43 in response to DiMNF, in both IGROV-1 cells (Figure 5(A)) and primary normal human bronchial epithelial (NHBE) cells (Figure 5(B)) with negligible cytotoxicity. Next, we tested the antiviral activity of DiMNF on ancestral and omicron SARS-CoV-2 strains at two different MOIs and did not observe any significant reduction in viral titres (Figure 5(C)). As AHR depletion was shown to reduce SARS-CoV-2 infection in IGROV-1 cells (Figure 4(C)), yet DiMNF treatment only reduced HCoV-OC43 replication but not SARS-CoV-2, the role of AHR in facilitating betacoronavirus infection warrants further investigation.

### DiMNF activates interferon stimulated genes in human primary bronchial epithelial cells

**Figure 6.**
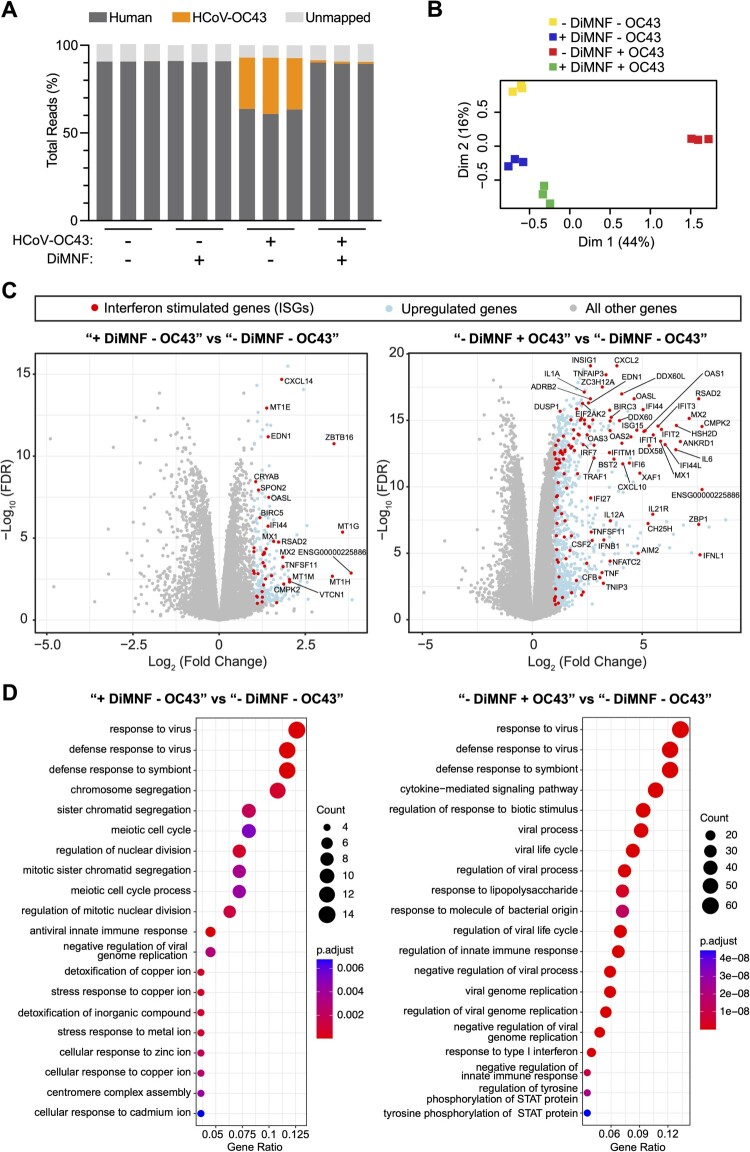
DiMNF treatment and HCoV-OC43 infection impact on normal human bronchial epithelial cells. (A) Proportion of all obtained reads getting aligned to human genome and HCoV-OC43 genome, determined by STAR aligner (*n* = 3). (B) Clustering of cells based on their overall gene expression count profiles, determined by MDS first two dimensions. Replicates from each condition are highlighted with different colours and are unsupervisedly clustered together. HCoV-OC43 infected cells treated with DiMNF are clustered closer to non-infected conditions. Values in parenthesis show the percentage of total variation captured in each of the first two dimensions. (C) Volcano plots of gene expression changes upon DiMNF treatment and HCoV-OC43 infection, individually. Upregulated genes are determined by Log_2_(fold-change) > 1 and Log_10_(FDR)<−1. (D) Biological processes gene ontology (BP) enrichment in the upregulated genes upon DiMNF treatment (left) and HCoV-OC43 infection (right). Over-representation analysis is done via clusterProfiler package in R, with multiple testing *p*-value adjustment by Benjamini-Hochberg method. Only top 20 statistically significant enriched entries are shown. Uninfected NHBE cells treated with DMSO are denoted as “- DiMNF - OC43.” NHBE cells infected with HCoV-OC43 and treated with DMSO are denoted as “- DiMNF + OC43.” Uninfected NHBE cells treated with DiMNF drug are denoted as “+ DiMNF - OC43.” NHBE cells infected with HCoV-OC43 and treated with DiMNF drug are denoted as “+ DiMNF + OC43.”

To reveal the underlying mechanism of action conferring the selective antiviral property of DiMNF, we performed bulk RNA sequencing transcriptomic analyses of primary NHBE cells infected with HCoV-OC43, in the presence or absence of DiMNF. The potency of the drug to mitigate HCoV-OC43 infection was again underlined by the diminished levels of viral RNA accumulation in infected cells treated with DiMNF (Figure 6(A)), as well as clustering of these cells with their uninfected counterparts in multidimensional scaling (MDS) analysis (Figure 6(B)). We observed a strong downregulation of canonical AHR-induced genes, especially *CYP1A1*, *CYP1B1*, and *MMP1*, in samples treated with the drug, confirming that DiMNF acts as an antagonist for the AHR canonical pathway [41–43] (Sup Figure 3(A)). Notably, we discovered upregulation of multiple ISGs, such as *ZBTB16*, *MX1*, *OASL*, *RSAD2*, *CMPK2*, and *CXCL14* in the DiMNF treated primary cells, even in the absence of viral infection (Figure 6(C)). This observation was recapitulated in the Gene Ontology over-representation analysis, demonstrating a basal activation of cell antiviral responses following DiMNF administration (Figure 6(D)). Consistent to the Gene Ontology analysis, several KEGG pathways related to cellular innate immunity were found to be significantly enriched in DiMNF treated cells (Sup Figure 3(B–E)). Of note, the strong induction of ISGs in response to HCoV-OC43 has not been investigated in primary human cells before, while a similar induction was observed in cell lines like Vero E6 [44]. As this basal upregulation of cellular innate immune genes upon drug treatment was only restricting HCoV-OC43 but had no impact on SARS-CoV-2 infection, our results suggest that other AHR downstream pathways, in addition to its known role in regulating innate immune response, are likely to simultaneously contribute to facilitating SARS-CoV-2 infection.

## Discussion

Several mammalian cell lines have been extensively used in SARS-CoV-2 studies, however, a consensus on the optimal *in vitro* system is not yet established. An optimal cell line would recapitulate the natural infection cycle of the virus, without requiring the addition of entry factors. Desirable technical features include availability, ease of culture, capability to propagate the virus in high yields, and development of CPE following infection. In this study, by analyzing both transcriptome and proteome data from the CCLE initiative, we identified IGROV-1 as a candidate cell line for isolation and propagation of human betacoronaviruses. A similar study to identify cell lines expressing SARS-CoV-2 entry factors was previously performed, but in contrast to ours, solely relied on the CCLE transcriptome data to identify different candidate cell lines [45]. In parallel and in agreement with our work, IGROV-1 cells have been used to successfully isolate SARS-CoV-2 Omicron strains from COVID-19 patients [46]. We rigorously compared IGROV-1 with other cell lines currently being used for SARS-CoV-2 research including Vero derivatives, highlighting the potential of IGROV-1 to be employed in various virological and serological assays. The long-standing observation of interferon deficiency in Vero cells has been traditionally viewed as a favourable attribute, as it facilitates higher viral titres production [47, 48]. However, this very characteristic renders them even more inadequate and misleading when it comes to studying the pathways underlying viral infections. We have shown that IGROV-1 cells are able to produce SARS-CoV-2 in titres similar to Vero E6, while not harbouring a compromised innate immunity. Theoretically, the human cell line could be used for the isolation and propagation of any ACE2-TMPRSS2 dependent viruses from clinical and animal samples, beyond known betacoronaviruses. Nevertheless, it is noteworthy that the present data does not entirely eliminate the potential of Vero cell derivatives in coronavirus research. More thorough examinations of IGROV-1 across diverse assays as well as a consensus from the coronavirology community would be essential before concluding that the human cell line could serve as a complete substitute for Vero cells.

Moreover, we have demonstrated that IGROV-1 is a useful tool to study the cell biology of betacoronavirus infections, through discovery of HCoV-OC43 and SARS-CoV-2 host dependency factors using the unbiased forward genetic screening strategy. AHR, the top host factor shared by SARS-CoV-2 and HCoV-OC43 in IGROV-1, was originally known to be a transcription factor regulating cellular response to various xenobiotic compounds [49]. It was later discovered that AHR also plays a pivotal role in modulating inflammation as well as the host cellular response to a variety of microbial pathogens [50–52]. In particular, AHR has been identified as a host dependency factor for a few enveloped viruses such as Dengue virus and Zika virus [53] – reviewed comprehensively in Hu *et al*. [54]. Moreover, Giovannoni *et al.* have recently reported that AHR is transcriptionally upregulated upon SARS-CoV-2 infection both *in vitro* and in patients samples [37], speculating that SARS-CoV-2 requires AHR to evade cellular antiviral responses, as previously reported for flaviviruses [53]. Additionally, while our manuscript was in preparation, Shi *et al.* (2023) reported AHR to be a pro-viral host factor for SARS-CoV-2 infection. In alignment to our results, they have similarly observed that the inhibition of the interferon response through JAK inhibitors proved inadequate for the restoration of SARS-CoV-2 infection in cells with AHR perturbation [38]. It is worth mentioning that both the abovementioned studies have utilized a commonly used AHR inhibitor, CH223191, to show chemical perturbation of AHR could potently inhibit all human betacoronaviruses. While the exact molecular mechanism leading to AHR activation upon betacoronavirus infection is yet to be determined, a previous report studying murine betacoronavirus MHV has suggested the mechanism to be IDO1 independent [55]. Serving as a critical cytoplasmic sensor of xenobiotic compounds, AHR can elicit different cellular responses appropriate to its diverse stimuli. This extends to modulators and inhibitors of the AHR protein as well, which, based on their distinct chemical interaction with the receptor, can induce dissimilar effects downstream of the receptor [56–58]. Using pinostrobin, another AHR inhibitor, Zhao *et al.* have proposed that regulation of fatty acid metabolism is also a contributing factor of AHR promoting HCoV-OC43 infection [59]. Utilizing DiMNF, an AHR antagonist that significantly diminished HCoV-OC43 infection but not SARS-CoV-2, we were able to interrogate AHR downstream pathways that might be contributing to SARS-CoV-2 infection. While AHR was previously known to be able to hinder several cellular innate immune genes, our results suggest that inhibition of such factors may not be the sole underlying mechanism to promote SARS-CoV-2 infection. It is also worth mentioning that the involvement of AHR in viral infections extends beyond the cells, encompassing broader implications at the tissue and organ levels. For instance, AHR has been reported to contribute to the pathogenesis of SARS-CoV-2 due to its role in mucus production, inducing hypoxia in COVID-19 patients [60]. More comprehensive approaches need to be employed in future investigations, including comparative transcriptomics, proteomics, and metabolomics analysis using a diverse array of AHR chemical inhibitors and genetic perturbations. These approaches are imperative to better understand the role of AHR in the modulation of betacoronavirus infection and pathogenesis. Given that AHR's engagement is multifaceted, encompassing intracellular and intercellular levels, it is equally crucial to validate these findings within animal models as well.

## Biosafety statement

All experiments involving the usage of live SARS-CoV-2 virus have been evaluated and approved by the Duke-NUS ABSL3 Biosafety Committee and the NUS Institutional Biosafety Committee. Regarding the PRNT experiments using convalescent sera, written informed consent was obtained from patients as part of a larger multicentre observational cohort study characterising emerging infectious diseases (PROTECT study, reference number 2012/00917). All human specimens were considered infectious and hazardous and handled using standard biosafety procedures.

## Supplementary Material

Supplemental Material

Supplemental Material

Supplemental Material

Supplemental Material

Supplemental Material

Supplemental Material

Supplemental Material

## Data Availability

All raw NGS datasets generated in this work are accessible publicly via Gene Expression Omnibus (GEO) under accession number **GSE237132**.
