# Supplementary figures and images for: Betacoronaviruses SARS-CoV-2 and HCoV-OC43 infections in IGROV-1 cell line require aryl hydrocarbon receptor
Source: Emerg Microbes Infect. 2023 Sep 6;12(2):2256416. doi: 10.1080/22221751.2023.2256416 (PMC10512916; doi:10.1080/22221751.2023.2256416)

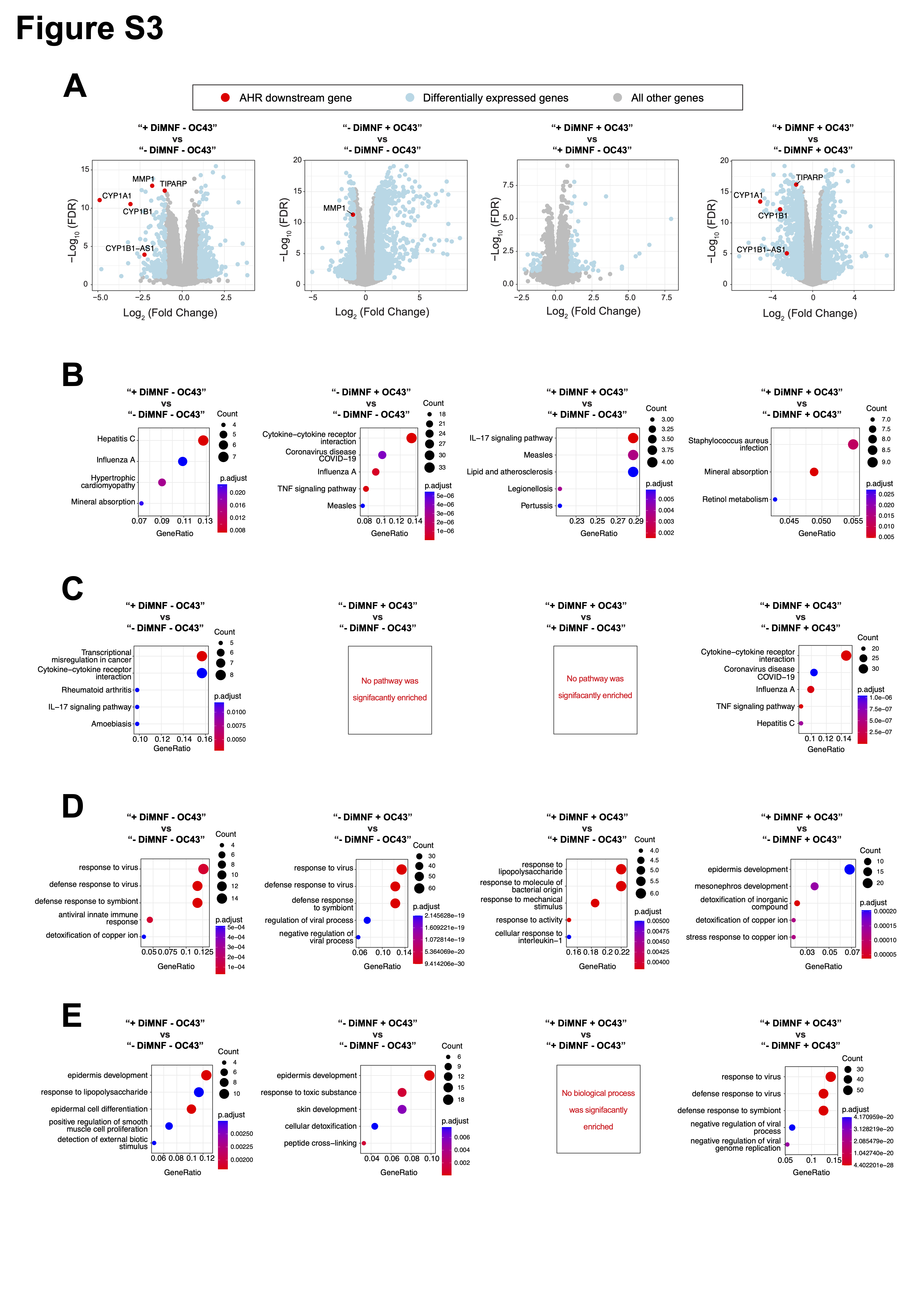

Supplement: Supplemental Material [file TEMI_A_2256416_SM8340.png]

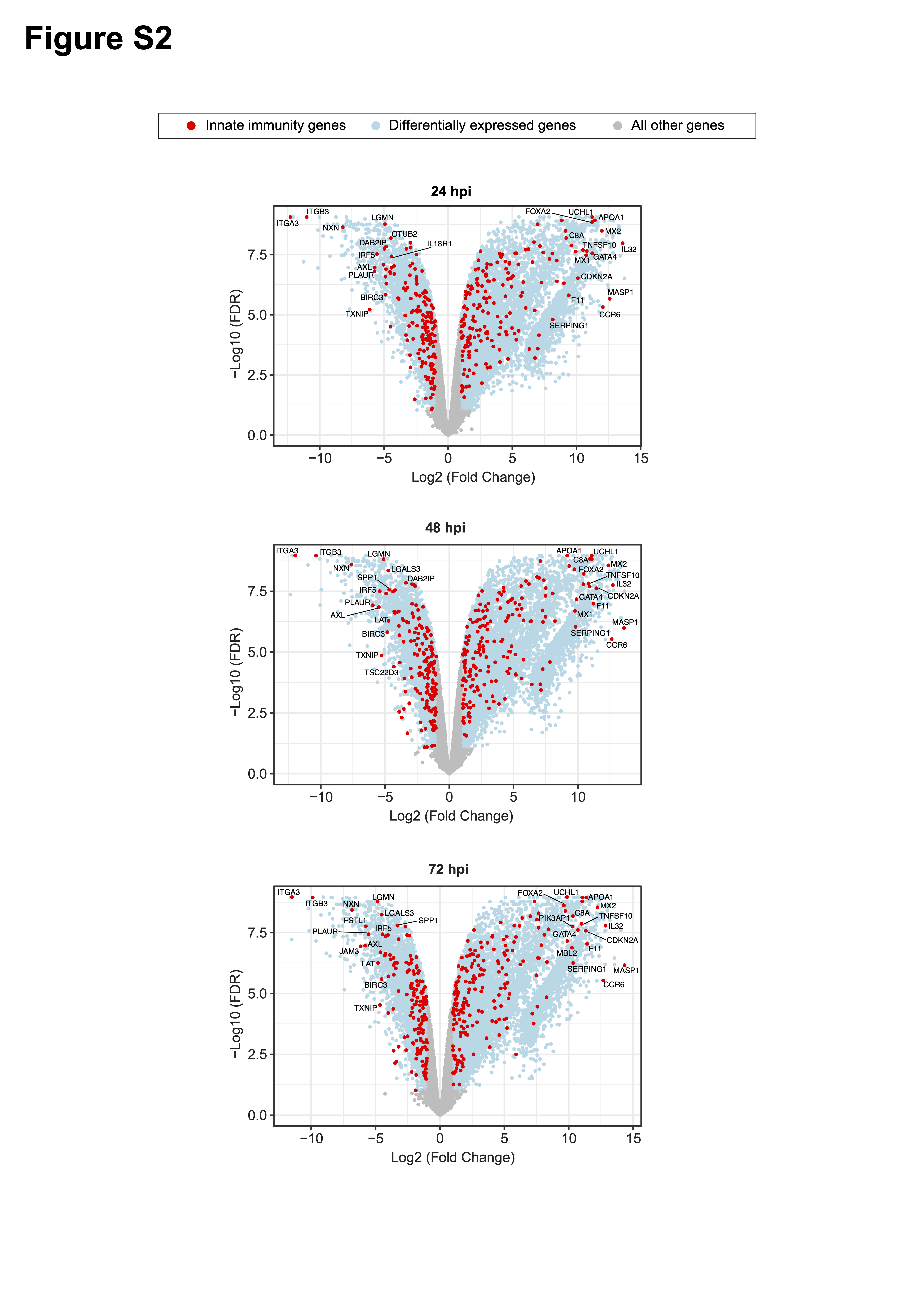

Supplement: Supplemental Material [file TEMI_A_2256416_SM8332.png]

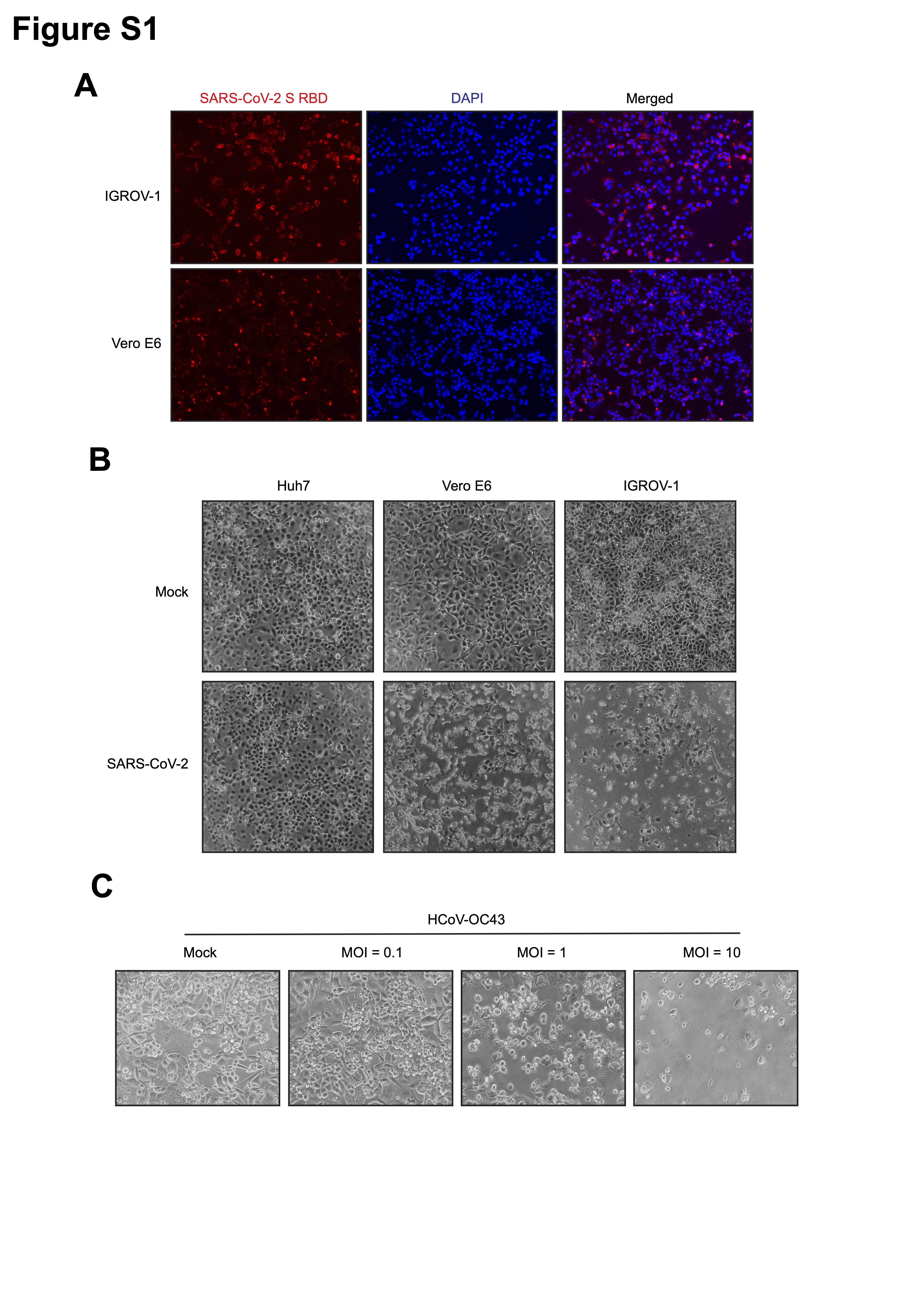

Supplement: Supplemental Material [file TEMI_A_2256416_SM8329.png]
